# MUC1 is a potential target to overcome trastuzumab resistance in breast cancer therapy

**DOI:** 10.1186/s12935-022-02523-z

**Published:** 2022-03-05

**Authors:** Aysooda Hosseinzadeh, Parnaz Merikhian, Nazanin Naseri, Mohammad Reza Eisavand, Leila Farahmand

**Affiliations:** Recombinant Proteins Department, Breast Cancer Research Center, Motamed Cancer Institute, ACECR, South Gandi, Vanak Squar, 1517964311 Tehran, Iran

**Keywords:** MUC1, Trastuzumab, HER2, Cancer, Drug Resistance

## Abstract

Although resistance is its major obstacle in cancer therapy, trastuzumab is the most successful agent in treating epidermal growth factor receptor 2 positive (HER2 +) breast cancer (BC). Some patients show resistance to trastuzumab, and scientists want to circumvent this problem. This review elaborately discusses possible resistance mechanisms to trastuzumab and introduces mucin 1 (MUC1) as a potential target efficient for overcoming such resistance. MUC1 belongs to the mucin family, playing the oncogenic/mitogenic roles in cancer cells and interacting with several other oncogenic receptors and pathways, such as HER2, β-catenin, NF-κB, and estrogen receptor (ERα). Besides, it has been established that MUC1- Cytoplasmic Domain (MUC1-CD) accelerates the development of resistance to trastuzumab and that silencing MUC1-C proto-oncogene is associated with increased sensitivity of HER2^+^ cells to trastuzumab-induced growth inhibitors. We mention why targeting MUC1 can be useful in overcoming trastuzumab resistance in cancer therapy.

## Introduction

Cancer is a type of disease in which cells proliferate out of the control of precise mechanisms, causing morbidity and mortality. In the normal cell, a tumor suppressor and mitogenic mechanisms act so that the cell does mitosis or apoptosis at the proper time; then, no cell proliferates at the wrong time. From a cellular point of view, several tumor suppressor mechanisms are switched off, and some mitogenic mechanisms are switched on in the cancer cells. Hence, cells proliferate uncontrollably, making a problem termed cancer. As the result of uncontrolled proliferation and tumor cell survival, the abnormal cell growth is augmented, representing a potential to invade or spread to other parts of the body [1, 2].

Before 1998, the HER2^+^ subtype of BC was worst in survival with a poor prognosis. However, trastuzumab (Herceptin), a humanized monoclonal antibody that targets the extracellular domain of HER2, has changed this situation [3]. After trastuzumab, the prognosis of the HER2^+^ subtype became better, and it has successful achievement in prolonging patients' life. Trastuzumab is categorized as a targeting drug since it targets specifically tumor cells that overexpress HER2 and block its activity.

Not only all of the HER2^+^ patients do not respond to Herceptin at the beginning, but also patients who respond initially become resistant within a year after treatment; the former is called primary, and the latter becomes drug-resistant [4–6]. It needs to be considered that not only diversity between patients is an obstacle, but also plasticity of cancer cells makes it difficult to find out the resistance mechanisms to Herceptin [7]. Even a single type of cancer may have heterogeneity in its gene profile expression.

Among several studies on how tumor cells escape from trastuzumab, compensation of signaling with other molecules plays a crucial role in resistance. Among these molecules, the role of the mucin family is step by step established. It seems that some mucin family members, such as MUC1, can trigger compensative signaling in HER2^+^ targeted cells and help them become resistant. Although, at first glance, a direct relation between MUC1 and trastuzumab resistance is not well clear, molecular studies reveal the complex network in which MUC1 plays a key role in trastuzumab resistance. In this review, we focus on the main mechanisms of trastuzumab resistance and discuss the vital role of HER2-MUC1 crosstalk in the resistance of BC cells.

## Trastuzumab-mechanisms of action

Since trastuzumab is an antibody, its mechanisms of action are categorized as immunologic and non-immunologic. The important immunologic mechanism is that antibody opsonizes tumor cells, and they become attractive to macrophages and natural killer (NK) cells for antibody- dependent cellular cytotoxicity (ADCC) mediation [8, 9]. By antibody binding, not only ADCC is triggered, but also several HER2 activities are prevented, blocking ligand-independent HER2 dimerization and inhibiting HER2-forming heterodimers with other EGFR family members [8, 10–12]. By endocytosis of HER2 and its intracellular degradation, HER2 expression is reduced [13–15].

When HER2 cannot form homo or heterodimers, its downstream signaling is dampened. Trastuzumab reduces AKT activity [10, 11] and increases P27 concentration in the nucleus. The inhibition of downstream activities leads to proliferation blockage, angiogenesis /deficiency in DNA repair, G1 cell cycle arrest, and apoptosis induction [8, 16]. How can it be possible that cancer cells withdraw all these action mechanisms?

### Resistance to trastuzumab

First, an antibody may interact with other proteins except for HER2. Moreover, shedding of HER2 epitope and high or low expression of HER2 on the surface of tumor cells before using reduce trastuzumab efficacy [8].

Resistance to trastuzumab mostly occurs due to incomplete inhibition of the PI3K/ mTOR pathway [17]. Loss and/or mutation of PTEN as a tumor suppressor gene results in overactivation of the PI3K/mTOR pathway [15, 18, 19]. Mutations in PIK3CA trigger signaling independently from HER2/HER3 heterodimer in trastuzumab resistant (Tras-R) cell [10]. Also, this pathway may be active constitutively, causing hyperactivation in mitogenic and survival signaling [8].

The most complex phenomenon occurring in resistant tumor cells is the crosstalk of HER2 and its downstream signaling with other receptors resulting in compensation in the signaling pathway and survival of tumor cells. For example, HER2 and HER3 can form a dimer with IGF-IR, another mitogenic receptor from a different family [4]. Abnormal IGF-IR activation is observed in several resistant tumor cells targeted by anti-EGFR therapies [11, 20].

Some mucins, such as MUC4, mask HER2 and do not allow trastuzumab interaction [11, 21]. Another example of this kind of resistance to trastuzumab is developed by MUC4, masking epitope part of HER2 and leading to the prevention of HER2/Trastuzumab complex formation [22]. In breast and gastric cancers, MUC1 expression plays a role in trastuzumab resistance; anti-MUC1 monoclonal antibody (mAb) overcomes this situation [5]. By MUC1 overexpression on the surface of BC cells, they would be resistant to the trastuzumab-mediated ADCC [23].

The results show that the mucin family is distinguished among different families that may crosstalk with HER2 receptor. The normal function of the mucin family is far from oncogenic activity. According to ongoing researches, the significant correlation between enhanced expression of MUC1 in tumor cells, as well as amplification of cancer cell proliferation and metastasis, is related to modulation of multiple signaling pathways [24–26]. This thereby suggests that the overexpression of MUC1 can potentially enhance transduction of inward survival signals and steer tumors toward resistance to trastuzumab in a malignant phenotype for progression. Here, at first, the role of mucins in tumorigenesis is discussed; then, it is focused on the important crosstalk of MUC1/HER2, promoting trastuzumab resistance in tumor cells.

### Mucin family

The mucin family contains 21 members, including transmembrane and secreted types. They all have specific tandem-repeat, serine-threonine, modified by O-glycosylation [27]. Both secreted and transmembrane act as a barrier for epithelial cells [27]. In a normal situation, the mucin family protects the downer layer; however, they can induce transformation into tumorigenesis and aberrantly be expressed in some cancers. Transmembrane mucins, including MUC1, MUC4, MUC13, and MUC16, all, except MUC16, are heterodimers with their cleaved subunits [27]. All are translated to a single poly-peptide and cleaved (in some cases auto-cleaved) into N-terminal and C-terminal subunits. These subunits form a complex with non-covalent bonds [27]. N-terminal subunit contains O-glycosylated-tandem-repeat and hangs on the extracellular surface by its connection to C- terminal subunit, embedded in a membrane. By releasing the N-terminal, the C- terminal subunit triggers inflammation signals [27].

MUC1 is expressed abundantly; hypo-glycosylated and apical localization is misplaced in human BC cells [28, 29]. Furthermore, in addition to transformation and loss of polarity in cancer cells, MUC1 is aberrantly overexpressed on the entire borders of greater than 90% of BC cells [30]. With the rise of trastuzumab resistance, many studies have been accomplished to delineate the underlying mechanisms involved in aberrant expression of MUC1-CD in acquired and intrinsic chemo-resistance. Thus, MUC1-CD has been well-established to associate with pathways, leading to trastuzumab resistance in HER2-overexpressing BC. In the following, the role of MUC1 in the growth and death of BC cancer cells is discussed.

### MUC, a proto-oncogene in BC cells

MUC1 is a proto-oncogene that interacts with all five members of the EGFR family, FGFR, PDGF [31], β-catenin, GSK3β, and EGFR [32]. It promotes transcription of genes, being involved in malignant phenotype in BC cells [33]. CQC motif of MUC1-CD is critical for homodimerization in cytoplasm and localization of MUC1 in the nucleus [34]. The MUC1 function would be lost if this motif is mutated or inhibited; for example, AQA mutation in this motif decreases AKT and ERK activation [35].

### Anti-apoptotic role of MUC1 in BC cells

Generally, MUC1 protects cancer cells from apoptosis. There are several mechanisms by which MUC1 inhibits apoptosis in cancer cells; MUC1 directly binds to the P53 regulatory domain^36^ and blocks apoptosis by interacting with P53 [37]. In BC cells, MUC1 and P53 interact in response to DNA damage [36]. Indeed, MUC1-CD reduces stress-induced cell death in BC cells [34]. Homodimers of MUC1 can localize in the nucleus and mitochondria [37]. More specifically, MUC1-CD is localized at the outer membrane of mitochondria and blocks cell death in response to DNA damage and oxidative stress [34]. By this mechanism, MUC1 suppresses DNA damage related to P53 independent and dependent apoptosis [36].

Another target of MUC1 is the BCL2 family. For example, MUC1 binds to BAX- pro-apoptotic in the BH3 domain and blocks its dimerization and BAX-mediated cytochrome C release [38]. In genotoxic stress, MUC1 interacts with BAX at mitochondria [38]. The mutation of the CQC motif blocks BAX/MUC1 interaction [38]. Another vital molecule is Bcl2A- anti-apoptotic, being overexpressed in MUC1-NF-κB manner in triple negative breast cancer (TNBC) cells [34].

MCL is an anti-apoptotic molecule, acting against pro-apoptotic molecules, such as BIM, BAD, and BAX [35]. Through post-translation modification of MCL, MUC1 upregulates MCL expression in BC cells [35]. MUC1 increases ERK activation, resulting in MCL phosphorylation and activation [35]. MUC1-CD increases MCL level through the mTOR and MEK/ERK pathway and enhances MCL's stability [34]. MCL activity makes cancer cells resistant to anti-cancer drugs [35]. By MUC1 silencing, activation of MCL significantly is decreased [35].

In fibroblast cells of rats, MUC1 activates the AKT pathway, increases BCLxl expression, and suppresses the intrinsic apoptosis pathway [39]. Furthermore, MUC1-CD increases the expression of anti-apoptotic protein, including BCLxl and MCL, in NF-κB dependent mechanisms in BC cells [34].

Besides, MUC1-CD interacts directly with caspases 8 and prevents its recruitment to death- inducing signaling complex (DISC). Hence, MUC1 can dampen death receptor-induced caspases 8 activity [5, 40, 41]. In cancer cells, MUC1 blocks cytochrome C, caspase 3 activity, and TRAIL-dependent apoptosis [42].

Involvement in the growth and death of cancer cells is not the only way that MUC1 promotes BC tumor progression. Studies have revealed that MUC1 might affect immune cells.

### Immunosuppressive role of MUC1 in BC

There are some facts that MUC1 suppresses the immune response and promotes tumor growth. First, cancer cells that overexpressed MUC1 are resistant to the cytotoxicity of T and NK cells, according to in vitro studies [43]. MUC1 helps immune invasion of BC cells by possible evading from lymphocyte-activated killer cells [44]. The results show that MUC1 directly inhibits T cell proliferation [31, 45] and induces apoptosis in Ag-activated T cells in BC [31].

PDL1 –CD274 is an inhibitor that suppresses the function of T cells. By inducing PDL1 expression through NF-κB and c-MYC activation in TNBC cells, MUC1 probably helps cancer cells escape from immune cells [46]. MUC1 promotes NF-κB and c-MYC localization in PDL1 promoters [46]. Moreover, suppression of MUC1-CD decreases PDL1 expression in such cells [46]. Another link between MUC1 and immune suppression is Cyclooxygenase-2 (COX2), which converts arachidonic acid to prostaglandin E (inhibitor of T and dendritic cells (DCs)). COX2 expression is related to advanced and large breast tumors and loss of the function of T and DC cells[47].

In the following, we survey the possible crosstalk of MUC1 with other molecules directly or indirectly involved in trastuzumab resistance.

### MUC1/EGFR family receptors interaction

The interaction of MUC1 forms heterodimer complexes with the four EGFR family receptors [48]. MUC1 can be another co-receptor of HER2 overexpressed in BC cells and other types of cancer [49]. When cells lose their polarity, MUC1 is expressed on the whole cell surface, and it can interact with HER2 [25, 30]; HER1 forms heterodimer [30, 50]. MUC1 is able to activate HER2 [51]; hence, it can be concluded that HER2/MUC1 relation is bilateral or in a positive loop manner [25].

Silencing MUC1 reduces HER2 activation, which is overexpressed cells [18, 52]. MUC1 has a specific site that facilitates the formation of the MUC1- HER2 complex [53, 54].

Biochemical interaction of MUC1 and EGFR in cell lines or mice models has been proved [32]. MUC1 stabilizes EGFR [55], inhibits EGFR degradation, and increases its internalization and recycling in BC cells [55]. YEKV is a motif of MUC1-CD, phosphorylated by EGFR [32]. HRG-induced activation of EGFR increases MUC1 localization at mitochondria [56]. Activated EGFR phosphorylates MUC1-CD at the YEKV motif, resulting in c-SRC biding [57].

Because MUC-CD has no N-terminal, Heat Shock Protein (HSP) 70 and 90 are associated with it [56]. In BC cells, by activation of EGFR, the c-SRC pathway is activated, promoting MUC1/HSP90 binding and MUC1 localization at mitochondria [56]. HRG-induced c-SRC activation phosphorylates Tyr 46 MUC1 and induces MUC1-HSP90 interaction [56].

### PI3K/AKT/mTOR/HIFα pathway/ MUC1 interaction

The PI3K/AKT/mTOR/HIFα pathway is a crucial signaling system with a significant role in proliferation, growth, and survival in normal cells [58]. Deregulation of this pathway is detected in over 70% of human BC cases [59], and it is a determinant signaling cascade involved in resistance to various targeted therapies [60], including trastuzumab treatment in HER2^+^ or endocrine treatment in ER^+^ BC [61]. This pathway is activated by tyrosine kinases, G-protein-coupled, or insulin receptor family and eventually results in the expression of genes, driving cellular proliferation and survival [62]. PI3K signaling initiates with relieved inhibition effect of the p85-the regulatory subunit of PI3K on p110-the catalytic subunit of PI3K molecule; then, p110 phosphorylates PIP2 and produce PIP3, which as a secondary messenger is activated the PI3K/AKT/mTOR/ HIFα cascade [63]. MUC1-CD is an anchor for binding of PI3K and consequent activation of the AKT pathway [35]. Indeed, the Y20HPM motif in the cytoplasmic domain of MUC1 interacts with p85 SH2 domains when phosphorylated on tyrosine and resulted in the activation of the PI3K/AKT/mTOR/ HIFα pathway. Furthermore, studies reveal that mutated MUC1-CD, which altered at Tyr-20 in the cytoplasmic domain of MUC1, contributes to activation of this cascade by preventing interaction between p85 and p110 in the PI3K molecule [64, 65]. Raina et al. show that blocking MUC1 is associated with a decreased level of p-AKT and cyclin E and response to trastuzumab combined with chemotherapy in resistant HER2^+^ cell lines [25]. In BC cells, MUC1 translation is increased by the AKT pathway, and MUC1 activates this pathway: auto-inductive loop [26].

### NF-κB/MUC1 interaction

**Fig. 1:**
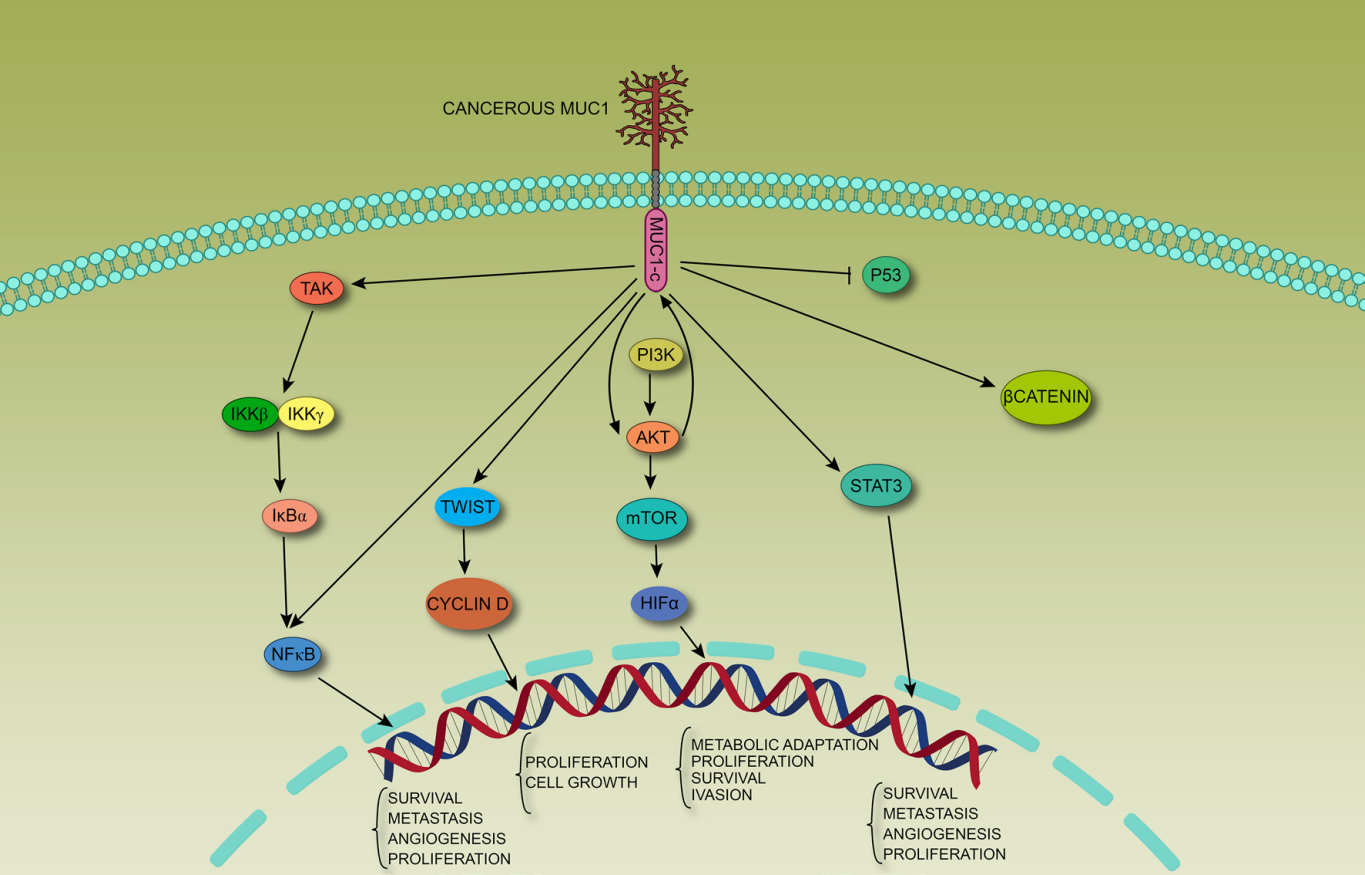
MUC1 interacts with several oncogenic pathways like NF-κB. It can activate TGFβ-activated Kinase (TAK) directly and activated NFκB pathway promotes survival and metastasis in BC cells. Besides, MUC1-CD is necessary for TWIST activation and it correlates to overexpression of cyclin D in BC cells. Moreover, MUC1-CD inhibits P53 activity in BC cells, thus it blocks apoptosis in these cells. MUC1-CD has wide range of interactions with different oncogenic and apoptotic molecules. In general, it promotes cell proliferation and resistance to apoptosis in BC cells

NF-κB is a multicomponent transcription factor with two major parts, including IκBs inhibitors of NF-κB and IKKs as the kinase complex, which in cooperation regulate expression of inflammatory proteins and development of mammary glands [66, 67]. MUC1-CD directly interacts with IKKβ and γ, activating the IKK complex (Fig. 1) [68, 69]. MUC1 directly binds to NF-κB and inhibits NF-κB/ IκBα interaction in BC cells [68, 70]. MUC1-CD directly interacts with NFκB, thus promoting NF-κB target gene expression and epithelial-mesenchymal transition (EMT) [34]. MUC1 helps TNBC cancer cells self-renew [71], probably through NF-κB (because NF-κB is linked to self-renew in BC cells) [52]. MUC1 silencing reduces NF-κB activity in cancer cells [69, 70]; it further interrupts the self-renewal of tumor cells [52]. By MUC1 silencing, NF-κB65 –induced TNFα activation is disrupted [69].

Deregulation of this pathway is related to overexpression of anti-apoptotic proteins such as Bcl-2 and BCLxl and progression in various types of cancers [72, 73]. The crosstalk between NF-κB and other pathways, such as ERK and AKT in HER2/EFGR signaling, leads to resistance to tyrosine kinase inhibitors in cancer cells IL6 via the NF-κB pathway link inflammatory responses to malignancy and generates an inflammatory feedback loop involved in trastuzumab resistance in HER2^+^ BC [74]. Approximately 200 amino acids sequence is needed for full overexpression of MUC1 in MCF-7 cell lines, being highly activated inside. This sequence has sites for NF-κB, STAT, and AP-3 bindings [75]. Some cytokines and peptide hormones regulate MUC1 expression through NF-κB and STAT3 [53, 75]. For example, TNFα R in the presence of IFNγ induces the binding of NF-κB to the MUC1 promoter [75]. MUC1-CD interacts with NF-κB and STAT3 in the nucleus [53], another positive loop between MUC1 and the mitogenic pathway.

### ERα/MUC1 interaction

Estrogen receptor α -ERα- is a ligand-regulated transcription factor critical in mammary gland tissue development [76, 77]. The aberrant expression of ERα is detected in more than 70% of patients with BC disease and leads to progressed tumor cells through transduced mitogenic action of estradiol [78]. Tamoxifen is a kind of selective ER modulator and acts as a decisive factor in reducing the rate of BC mortality in recent years [79].

MUC1 oligomerization inhibitor blocks BC tumor growth, dependent and independent of estrogen [80]. Molecular studies show that MUC1-CD also links to ERα in the MCF-7 cell line [81]. MUC1 and ERα are co-expressed with similar expression levels in the MCF-7 cell line. Estrogen induces ERα-MUC1 direct interaction [81]. The ERα- MUC1relation is not just limited to their physical contact; MUC1 regulates ERα translation. MUC1 binds estrogen-responsive gene promoter and also recruit its related co-activators [81]. Estrogen-induced MUC1 and ERα inhibitor (ICI) blocked MUC1 expressions induced by estrogen can bind MUC1 promoter [82]. After the accumulation of MUC1-CD in the cytosol, it translocates to the nucleus and binds to ERα. ERα- MUC1 complex recruits to ER promoter and facilitates its translation [83, 84].

Some interesting findings prove the intimate relation of ERα- MUC1; by silencing MUC1 (MUC1-siRNA), estrogen does not affect MCF-7 cell line growth [81]. MUC1-CD not only affects ERα expression but also can directly bind to DBD-ERα and stabilize it [26, 81, 85]. ERα is stabilized by MUC1 through blockage of ERα degradation [26]. Moreover, MUC1 induces tamoxifen resistance cells by interacting with HER2, thereby activating PI3K/AKT/mTOR in ER^+^ BC [86].

### β-Catenin/MUC1 interaction

WNT/β-catenin pathway signaling has a key role in metastasis [87], resistance to chemotherapy, and radiotherapy in cancer cells [88]. WNT/β-catenin singling is activated by binding the WNT ligand to Frizzled (FZD) receptor family, and then FZD, in turn, binds to its co-receptor lipoprotein receptor-related protein6 (LRP6) or LRP5 [89]. WNT-FZD-LRP complex prevents phosphorylation and degradation of β-catenin; in the next step, stabilized β-catenin is translocated to the nucleus and forms a transcription complex with TCF/LEF [90]. β-catenin in the nucleus acts as a transcriptional co-activator and results in the expression of genes essential in cell proliferation and cell fate determination [89]. MUC1/β-catenin interaction severely induces metastasis [91]. MUC1 overexpression disrupts cell–cell junction and cell–matrix adherent and promotes metastasis [43].

Another source of β-catenin is detected in the membrane, and it is participated in adherent junction by linking E-cadherin to the cytoskeleton. β-catenin, E-cadherin, and MUC1 form a complex, decreasing cell junction, thus increasing the migration of cancer cells [37]. Specific sequence (SXXXXXSSL motif) of MUC1-CD can interact with β-catenin; this interaction in the nucleus leads to the transcript of EMT genes [92]. In addition, overexpressed MUC1 is able to compete E-cadherin for β-catenin binding [48, 93–96]. When β-catenin interacts with MUC1, its interaction with E-cadherin decreases; hence, the cell can migrate [30, 48]. It can be concluded that MUC1 overexpression plays a role in cancer metastasis. The growth factor promotes MUC1 phosphorylation and its interaction with SRC and β-catenin expression and activity y [95]. Besides, MUC1 increases cancer cells' binding to endothelial cells through the expression of E-selection [43].

MUC1-CD also can interact with GSK3β in the STDRSPYEKV site; in turn, this interaction prevents forming β-catenin/MUC1 complex by GSK3β phosphorylation. Interaction between MUC1 and EGFR in the YEKV motif on MUC1-CD is associated with an increased level of β-catenin/MUC1 complex activity in the nucleus [97]. The same effect is detected about PKCδ, increasing interaction between MUC1 and β-catenin by phosphorylation in TDR site on MUC1 cytoplasmic tail [97, 98]. Phosphorylation of the YEKV motif leads to an increase of MUC1- β-catenin binding [57]. In metastatic BC patients, the MUC1- β-catenin interaction is significantly increased [91].

### STAT3/MUC1 interaction

Since trastuzumab is approved for breast and gastric cancers, their cell lines are investigated for resistance widely. Studies show that STAT3 is over-activated in gastric and breast Tras-R cell lines [18]. STAT3 is activated constitutively in several cancers; it is a mediator of cytokines and growth factors to the nucleus, promoting invasion, metastasis, angiogenesis, survival, and proliferation in cancer cells [99, 100]. It is activated mainly by EGFR family members in cancer [101]. In Tras-R BC cells, STAT3 is hyperactive; this situation is sufficient for the induction of Tras-R [18]. EGF is one of the STAT3 upstream activators, and MUC1 is STAT3 downstream targets [18]. STAT3 is a cytoplasmic protein, being translocated in the nucleus, acted as a transcription factor when phosphorylated on tyrosine residue, and then initiated to upregulate various proteins, including P53, MCL, BCLxL, cyclins D1/D2, c-MYC, VEGF, with a key role in oncogenesis [100, 102, 103]. MAPKp38, JNK, and mTOR activate STAT-3 maximally [100]. STAT3 interacts with HIFα and facilitates HIF-target gene transcription such as VEGF [101]. In BC cells, STAT3 increases MMP9 mRNA and protein levels [101].

Activation of STAT3 is detected in half of BC cases and inhibits expression of cytokines and chemokines, which possess pro-inflammatory function, thus suppressing immune cell activation. The synergy between p-STAT3 and IL6 in HER2^+^ BC promotes EMT and cancer stem cells proliferation; it is associated with trastuzumab resistance [103]. STAT3 is downstream of IL8 and IL35, after activation of which, STAT3 is activated to promote proliferation in BC cells [104].

MUC1 and MUC4 are two target genes of STAT3, with a remarkable role in trastuzumab resistance. In other words, IL6 activates STAT3, which is associated with the increased level of MUC1 and MUC4 expression (18, 74). STAT3 promotes MUC1 transcription [105]; on the other hand, MUC1 activates STAT3 and TWIST, as well as facilitating EMT [104].

### RAS/MAPK pathway/ MUC1 interaction

**Fig. 2:**
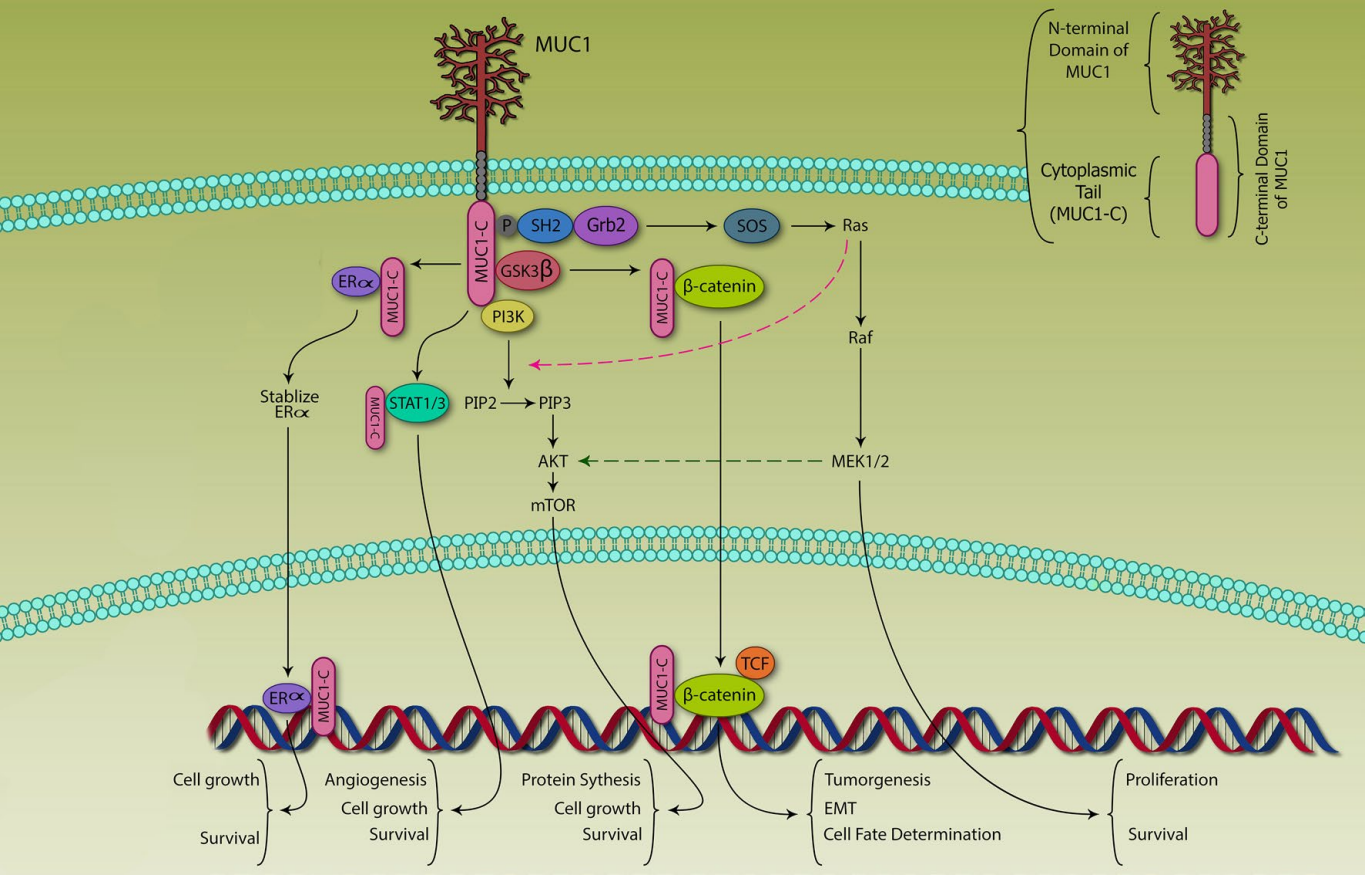
The intracellular portion of MUC1-CD can interact with multiple signaling molecules such as PI3K p85 subunit and GRB2, activating AKT-mTOR and RAS-RAF-MEK1/2 pathways. The cytoplasmic MUC1-CD can be further translocated into nucleus, where it associates with multiple transcriptional factors or nuclear receptors, such as β-catenin and ERα inducing the expression of targeted genes that are important to tumor cell proliferation or survival

Based on the novel data, the activation of MAPK is strongly correlated to overexpression of MUC1 proto-oncogene in aggressive BC cells [106]. One mechanism of MAPK activation is the recruitment of the Grb2/SOS complex, resulting in RAS, RAF, MEK, and ERK1/2 phosphorylation (Fig. 2) [48]. Activation of ERK1/2 can lead to their translocation into the nucleus and induction of gene transcription involved in cell proliferation and survival [107]. Indeed, their function as transcription factors in the nucleus regulates the expression of several genes involved in cell growth, differentiation, proliferation, survival, migration, and resistance development in the tumor.

As MUC1 overexpression enhances activation of the MAPK pathway through its cytoplasmic domains and decreases cellular adhesion, it is tempting to speculate a significant role for MUC1/EGFR signaling in interruption of the tight junction complex and MUC1-associated tumorigenesis [108, 109].

### Current approaches for overcoming trastuzumab resistance

As illustrated above, MUC1 is a proto-oncogene and shows crosstalk with several other oncogenic molecules and pathways involved in proliferation, survival, metastasis, and invasion. Because cancer cells use different mechanisms to become resistant to trastuzumab, there are several potential targets for overcoming trastuzumab resistance in such cells. Here, we briefly survey possible approaches that surrender Tras-R cells. Furthermore, Table1 provides examples of clinical trial studies targeting different signaling pathways that participate in trastuzumab resistance in BC.

**Table 1:** Examples of signaling pathways which are targeted in clinical setting for cancer treatment

Cancer	Target	Drug	phase	NCT ID
Breast, Head and Neck Squamous Cell Carcinoma, Non Small Cell Lung, Hepatocellular, Colorectal, Gastric Melanoma cancer	STAT3	TTI-101	I	NCT03195699
Solid Tumor, Pancreatic, Breast, Ovarian cancer	Stat3/NF-kB/	Imx-110	I, II	NCT03382340
Oestrogen Receptor Positive Advanced Breast Cancer	mTOR, ER	Exemestane, Everolimus	IV	NCT01743560
Estrogen-receptor Positive Invasive Metastatic Breast Cancer	JAK, ER	Ruxolitinib, Exemestane	II	NCT01594216
Estrogen Receptor-Positive, HER2-Negative Locally Advanced or Metastatic Breast Cancer	ER	Giredestrant, Letrozole	III	NCT04546009
Breast Cancer	Protein Kinase	Dasatinib	II	NCT00371345
Postmenopausal Metastatic Breast Cancer	ER, p38 MAPK	Tamoxifen, Ralimetinib	II	NCT02322853
Breast Cancer	ER, MEK1, MEK2	Fulvestrant, Selumetinib	II	NCT01160718
Breast Cancer Stage IV	ER, MEK1, MEK2	Mirdametinib, Fulvestrant	I,II	NCT05054374
Melanoma, Breast cancer	MEK	PD-0325901	I,II	NCT00147550
Breast, Colorectal, Head and Neck, Lung, Melanoma, Ovarian, Pancreatic, Prostate Cancer	26S proteasome/NF-κB /MAPK	Bortezomib, Paclitaxel	I	NCT00667641
Breast Cancer	TKR, aromatase, IGFIR	OSI-906, Erlotinib hydrochloride, Goserelin, Letrozole	II	NCT01013506
Breast, Non Small Cell Lung, Melanoma cancer	MEK1,2, PI3K and mTOR	MSC1936369B (pimasertib), SAR245409	I	NCT01390818

Reference: www.clinicaltrial.gov

In our previous paper, we discussed that pan-HER targeting might decrease tumor resistance to EGFR targeted therapy [12] because, as mentioned above, EGFR receptors show crosstalk with HER2, which plays key roles in resistance in Tras-R cells [110]. Here are some examples of targeting HER2 and HER3 in clinical/preclinical settings. Using pertuzumab (that blocks HER2 dimerization) combined with trastuzumab significantly has higher anti-tumor activity than trastuzumab alone [111]. In the preclinical setting, siRNA-HER3 combined with trastuzumab is used in BT474 (HER2^+^ BC cell line), inducing G1 arrest in these cells [110]. Another example is the ertumaxomab, a trifunctional bispecific antibody that targets CD3 and HER2 and is able to circumvent trastuzumab resistance [111]. Moreover, targeting HER2 and HER3 with pertuzumab and patritumab can overcome trastuzumab resistance, which is more efficient than each alone [112]. Another agent is MM-111 that targets HER2 and HER3, which suppresses tumor growth even in the presence of HER3 ligand in vivo when combined with trastuzumab [113]. Drug conjugate T-DM1 is another approach to overcome trastuzumab resistance. Since this agent is an antibody, it mediates ADCC and other antibody-mediated anti-tumor mechanisms; furthermore, its drug conjugate –emtansine- can directly show cytotoxicity against tumor cells [114].

For targeting crosstalk in receptor level agents, including lapatinib, which inhibits tyrosine kinase receptors, it is used combined with trastuzumab [111, 115–118]. In one study, in phase III clinical trial, lapatinib plus trastuzumab is more potent in tumor suppression than each agent alone [119]. Another target is IGF-IR and cixutumumab, a mAb, which targets IGF-IR in phase II clinical trial in BC patients treated with trastuzumab [120]. Anti-VEGF mAb, such as bevacizumab, in combination with trastuzumab, is in phase II clinical trial in BC patients [121].

One way is to target pathways, such as PI3K/ AKT/mTOR, downstream of several growth factor receptors, including EGFR family members [103]. There are some preclinical and clinical studies on PI3K [122], mTOR [123, 124], AKT [125], dual p70S6K/AKT inhibitors in phase I clinical setting [126] for defeating trastuzumab resistance in cancer cells. For example, several studies have tested the effect of everolimus –mTOR inhibitor- combined with trastuzumab in BC patients [127, 128].

Inhibition of HSP90, which stabilizes and promotes maturation of HER2, is another approach to circumvent trastuzumab resistance [129–131]. For example, tanespimycin, the inhibitor of HSP90, combined with trastuzumab in phase II clinical trials in BC patients, shows an anti-tumor effect [131]. On the other hand, targeting immune checkpoint CTLA-4 and PD-1 with mAbs is another approach for overcoming trastuzumab resistance [132, 133]. These molecules suppress immune system response, and targeting them boosts anti-tumor immunity.

### MUC1 as a potential biomarker for trastuzumab resistance

A study has shown that all BC circulating cells express MUC1 [134]; moreover, in HER2^+^ and TNBC subtypes of BC, MUC1 expression is significantly correlated to poor prognosis in patients [37, 135, 136]. Thus, scientists believe that MUC1 is a trastuzumab resistance marker [68]. The elevated level of soluble MUC1(sMUC1) is found in peripheral blood of BC patients [45]. CA 15.3 (MUC1 serum marker) can be a poor prognosis marker in ER^+^ and/or PR^+^ BC subtype [137]. Besides, MUC1 expression is negatively correlated to overall survival and relapse-free survival [137]. MUC1 can be a marker for BC diagnosis because its expression is significantly higher in cancer tissue than in normal tissue [137]. Indeed, the measurement of CA 15.3 can be useful in predicting response to trastuzumab [138]. The assay of sMUC1 was approved by FDA for disease monitoring in BC patients [45]. Several studies demonstrate that the elevated level of CA 15.3 in serum is significantly correlated with tumor size and metastasis in BC patients [138].

### MUC-targeting strategies

Facts support the idea that anti-MCU1 immunotherapy strategies would be useful in BC patients. For example, in the early stages of BC, patients with MUC1 natural antibodies might have less metastasis [139]. The correlation between natural IgG-MCU1 and improved overall survival is observed in these patients [140].

Data demonstrate that MUC1 is an ideal antigen for immunotherapy. First, it is expressed on the surface of the breast tumor [47]. Second, cancerous MUC1 is hypo-glycosylated, indicating that its core- the main antigen—is exposed [47]. Third, cancerous MUC1 has a different structure from normal MUC1, which is overexpressed only on cancer cells. The fourth and final, specific MUC1 sequence (PDTRP) is one of the most immunogenic epitopes of MUC1, which is the target of SM3 mAb [141].

Data show that MUC1 can be another target, which should be added to target molecules to restore trastuzumab sensitivity since it not only has crosstalk with several oncogenic/mitogenic pathways but also has a role in trastuzumab resistance in BC cells [138, 142]. Hence, MUC1 is a good target for mAb, vaccines, and inhibitors [141, 143]. For example, studies show that using siRNA MUC1 or anti-MUC1 mAb would make BC cells susceptible to trastuzumab-mediated ADCC [23]. In the following, we list several strategies that can be used for MUC1 targeting in BC therapy, including immunotherapeutic and non-immunotherapeutic strategies.

## Anti-MUC1-immunotherapeutic strategies in BC

### Monoclonal antibody

In the Phase-I clinical trial, the anti-MUC1 antibody- AS1402- is tested in metastatic BC patients. These patients have been previously treated and became resistant to taxol or anthracycline and could tolerate this antibody [85]. A continuous study has tested this antibody in combination with letrozole in metastatic BC patients in the phase-II clinical trial. Results have shown no positive effect on these patients [144]. Moreover, scientists have designed anti-MUC1 scFV, which could bind to MUC1-expressing BC cells and block their invasion and survival [37].

### Vaccine

A flagella vaccine that targets MUC1N has been tested in the mice model equal to stage IV of human BC, suppressing metastasis in these animals [145]. Moreover, the Sialyl-Tn vaccine is tested in metastatic BC patients in the clinical setting; it does not improve the survival of such patients [146]. Furthermore, metastatic BC patients are tested by PANVAC –MUC1and CEA vaccine- in the clinical setting, showing positive effects [147]. Other MUC1 and CEA vaccines have been tested in metastatic cancer patients, showing them to be safe and induce anti-tumor immunity [148]. L-BLP25 is a peptide vaccine that targets MUC1 and CEA and is under investigation in BC patients in the clinical setting [149].

### CAR T cell therapy

Chimeric antigen receptor T cells (CAR T) engineer T cells, which its T cell receptor contains scFV-from specific tumor Ag-Antibody- a transmembrane domain and a signaling domain [150]. CAR T cell setting has been used for targeting MUC1 in cancer, too. TAB004 –anti-MUC1 mAb- is used to produce CAR T cells. Zhou, Ru et al. show this strategy to be useful in inducing anti-tumor immunity in the TNBC mice model, with a cytotoxicity effect on the TNBC cell line [150].

### Combination immunotherapy

Many scientists believe that targeting one antigen or molecule is not as effective as they expect for cancer therapy. Thus, they decide to design a combination of different immunotherapies in such therapy. For example, the MUC1 peptide vaccine combined with COX1,2 inhibitor- indomethacin- significantly decreases tumor size in BC mice models [47]. In another study, DC vaccine fused with MUC1-mRNA in combination with anti-CTLA4 mAb -9D9- has increased anti-tumor immunity in TNBC mice models [151]. Silencing MUC1 combined with trastuzumab could only decrease AKT phosphorylation and induce apoptosis in Tras-R cells [18].

### Non-immunotherapeutic MUC1 targeting strategies

GO-201, 202, and 203 target the CQC motif of MUC1c and consequently blocks MUC1-CD homodimerization and its related function [34]. In one study, GO203 has suppressed HER2 activity [52] and blocked tumorigenesis in BC cells [35]. GO203 decreases BCL2A and MCL mRNA and protein levels in TNBC cells [34, 35]. GO201 and GO202 block MUC1 oligomerization and transduction to the nucleus and mitochondria and stop BC growth [37, 80]. GO201 blocks MUC1 functions such as keeping the reactive oxygen species balance in cancer cells [80]. Furthermore, GO201 induces cell cycle arrest at the S phase and blocks ERα dependent and independent tumorigenesis [80]. It also blocks NFκB/MUC1 interaction and NF-κBactivation in BC cells [70].

Using PMIP- a peptide- that targets MUC1 significantly decreases tumor growth in the mice model [32]. PMIP blocks MUC1- β-catenin co-localization in the surface of BC cells, and it hinders their invasion in vitro [32]. C-MET, in cooperation with MUC1 and EGFR, promotes EMT, migration, and invasion. PMIP reduces c-MET expression in BC cells [152].

### Aptamer

Aptamers are single-strand DNA or RNA binding to their targets with high affinity. They have low molecular weight and size and high penetration ability to the tumor; also, they are not immunogenic [153]. Aptamers are under investigation for cancer therapy, and MUC1 aptamer could recognize MUC1-expressing TNBC cells well [153]. DNA aptamer of MUC1 has been tested in BC cells, suppressing their growth [154]. Moreover, this aptamer reduces tumor growth in vivo [154].

### siRNA

Silencing MUC1 by siRNA in BC cells increases H2O2 level and increases sensitivity to oxidative stress [155]. It decreases HER2 activity in HER2 overexpressed BC cells [25]. siRNA-MUC1 could restore sensitivity to trastuzumab in gastric-trastuzumab resistant cells [80].

### Combination therapy

The effect of GO203 and taxol has been tested in MCF7 cells. Results have shown that these agents could induce nearly 35% G2/M cell cycle arrest and caspase 7 activation in cancer cells, more than each agent alone [156]. In a study, the combination of anti-MUC1 mAb-GP1.4- and the inhibitor of AKT and ERK'1/2 has been tested in BC cells [157]. This treatment has decreased BC MMP2 and 9 expressions and cell survival and induced G2/M cell cycle arrest and apoptosis [157]. Combining anti-MUC1 mAb –GP1.4- and platinum robustly induces apoptosis in MCF7 cells [158].

## Future perspective

From the immunological point of view, MUC1 targeting alone might induce anergy in T cells due to our body's natural tolerance to self-antigens. Hence, using potent immunotherapy settings such as targeting immunomodulatory molecules and MUC1 might be effective in this case. Besides, non-immunological approaches such as MUC1 siRNA or inhibitors can be added to the setting above. Also, MUC1 can be targeted indirectly; an agonist of aryl hydrocarbon receptor -I3C- decreases MUC1 mRNA in BC cells. Moreover, MUC1-targeting drugs and trastuzumab can be combined and used in BC therapy. By doing so, the chance of trastuzumab resistance possibly would be decreased. These are plausible approaches of MUC1 targeting in BC therapy that can be investigated in future experimental and clinical research.

## Conclusion

Studies have reported that MUC1 confers BC cell resistance via inhibition of pro-apoptotic properties and continuing activation of mitogenic pathways. MUC1 interacts with different mitogenic molecules and activities them; through its interaction with pro-apoptotic molecules, MUC1 inhibits them and blocks apoptosis. These mitogenic and pro-apoptotic molecules somehow play roles in trastuzumab resistance. Results of several in vitro and in vivo studies, which target MUC1 in BC cells, suggest that targeting MUC1 can restore trastuzumab sensitivity in BC cells or BC animal models. Hence, we propose that MUC1 is a potential target for overcoming trastuzumab resistance in BC therapy.

## Data Availability

Not applicable.
